# Are Regulation and Innovation Priorities Serving Public Health Needs?

**DOI:** 10.3389/fphar.2019.00144

**Published:** 2019-03-08

**Authors:** Christopher-Paul Milne, Kenneth I. Kaitin

**Affiliations:** Tufts Center for the Study of Drug Development, Tufts University, Boston, MA, United States

**Keywords:** new active substance (NAS), pharmaceutical R & D, innovation, drug development, regulatory agency

## Abstract

A host of challenges confront healthcare authorities worldwide. Topping the list is the demand for innovative new medicines to treat a range of both infectious and non-communicable diseases, while containing spiraling healthcare costs. The challenge is particularly great in therapeutic areas where, despite significant medical need and economic impact, the technical challenges and commercial risk of development serve as disincentives to drug sponsors. These areas include cardiovascular diseases as well as diseases and disorders of the central nervous system. Currently, the development and approval of new active substances, with its disproportionate focus on oncology, is not in alignment with healthcare needs in most geographic regions. In this article, we discuss the origins of this misalignment and suggest various approaches to address healthcare needs going forward.

## Are New Active Substance Launches Meeting Society's Needs?

Across the globe, spending on medicines as a percentage of overall healthcare expenditures ranges from 5 to 10% in most developed countries to as much as 60% in many emerging economies^1^. Despite the differences, healthcare systems are confronting the same dual challenges of controlling healthcare costs and the critical need for breakthrough treatments. Decision-makers must not only maintain adequate incentives for biomedical innovation, they must also ensure that the new medicines resulting from that innovation are accessible and affordable to patients who need them.

**Figure 1:**
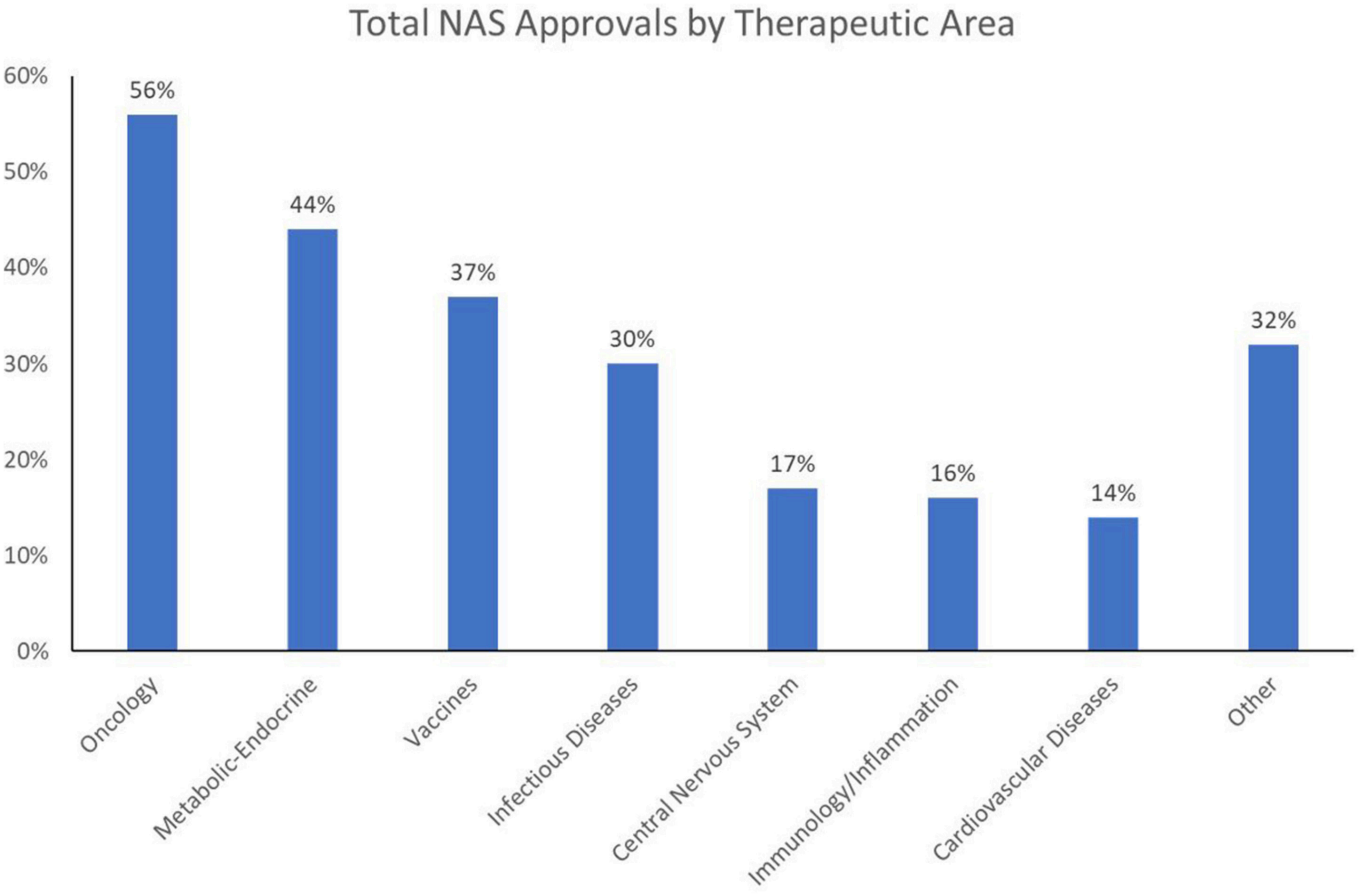
2013–2017 NAS approvals by therapeutic area.

These challenges are increasing in scope and complexity as the world tackles what the World Health Organization (WHO) refers to as the “double burden of disease”: i.e., the current crisis of emerging and re-emerging infectious disease epidemics and pandemics, and the growing impact of non-communicable diseases (NCD) on overall mortality and morbidity. Of 56.9 million global deaths in 2016, 40.5 million (71%) were due to NCDs: in particular, cardiovascular (CV) diseases (17.9 million, or 44% of all NCD deaths), cancers [9.0 million (22%)], and respiratory diseases, including asthma and chronic obstructive pulmonary disease [3.8 million (9%)]. Diabetes caused another 1.6 million deaths. Over three-quarters of NCD deaths−31.5 million—occurred in low- and middle-income countries, with about 46% of those deaths occurring in individuals 70 or younger (WHO, 2018). Currently, healthcare expenditures are an average of 4–5% of GDP in China and India—about half the amount spent in Western Europe and North America. Compounding the challenge is the fact that whereas prescription drugs are often considered one of the most cost-effective forms of medical treatment, the worldwide output of New Active Substances (NAS: the first approval of novel drugs anywhere in the world) has been limited in the range of unmet medical needs being addressed; over the 5-year period 2013–2017, just two therapeutic areas—oncology and infectious diseases—have dominated NAS launches worldwide (Figure 1)^2^.

## Are Industry Trends Helping or Hurting?

Oncology approvals have become dominant over the last decade. There has also been a surge in approvals in the infectious disease/vaccine (ID) area in recent years, due in part to heightened public awareness of global pandemics and antibiotic resistance. In contrast, approvals of new CV and central nervous system (CNS) agents have fallen far behind, a cause for concern for two reasons. The first is that these trends are not in sync with public healthcare needs. While cancer is certainly a major health issue, it is not the primary health concern in terms of mortality and morbidity; in the US and Western Europe, CV disease (CVD) is number one in overall mortality, and in many emerging and developed markets alike, CVD is associated with growing levels of morbidity and premature death. The second reason for concern is that the NAS approval trends run counter to the mission of national regulatory authorities. These authorities are tasked with addressing medical needs by dedicating energy and resources proportionate to the public health impact of the causative disease. When this is not done, agency decision-making on priorities and resource allocations should be re-evaluated, and recalibrated if necessary.

Current NAS approval trends are troubling in an additional context. While national regulatory authorities influence how many and how fast products reach the marketplace, it is the pharmaceutical industry that typically controls what types of drug candidates enter the development pipeline. The two therapeutic areas that have remained static in recent decades—CNS and CV—represent areas with substantial market potential. Mental health was tied with cancer as one of the four most costly medical conditions in the US during the decade of the 2000s, and the American Heart Association estimates that over a third of Americans currently suffer from some form of CVD. Worldwide, CVD is considered the fastest growing NCD health threat. For example, obesity has reached epidemic levels in some developing countries, as the populations have developed a growing penchant for western-style diets that pre-dispose to metabolic syndrome and its disease sequelae. In the CNS area, the WHO projects that by 2020, depression will be the second leading cause of disability worldwide (World Health Organization, 2004).

Despite the enormous market opportunity in the CV and CNS space, the number of NAS approvals in these areas is static or declining; CV and CNS combined equal only about half the number of oncology approvals in 2013–2017. Whereas, the recent dominance of oncology approvals is largely a US phenomenon (82% of oncology launches among global NASs from 2013 to 2017 were in the US), the facts that 58% of NASs worldwide originate in the US (148/256), and 47% of the worldwide pipeline is focused on oncology/immunology^3^, highlight a global concern going forward.

**Figure 2:**
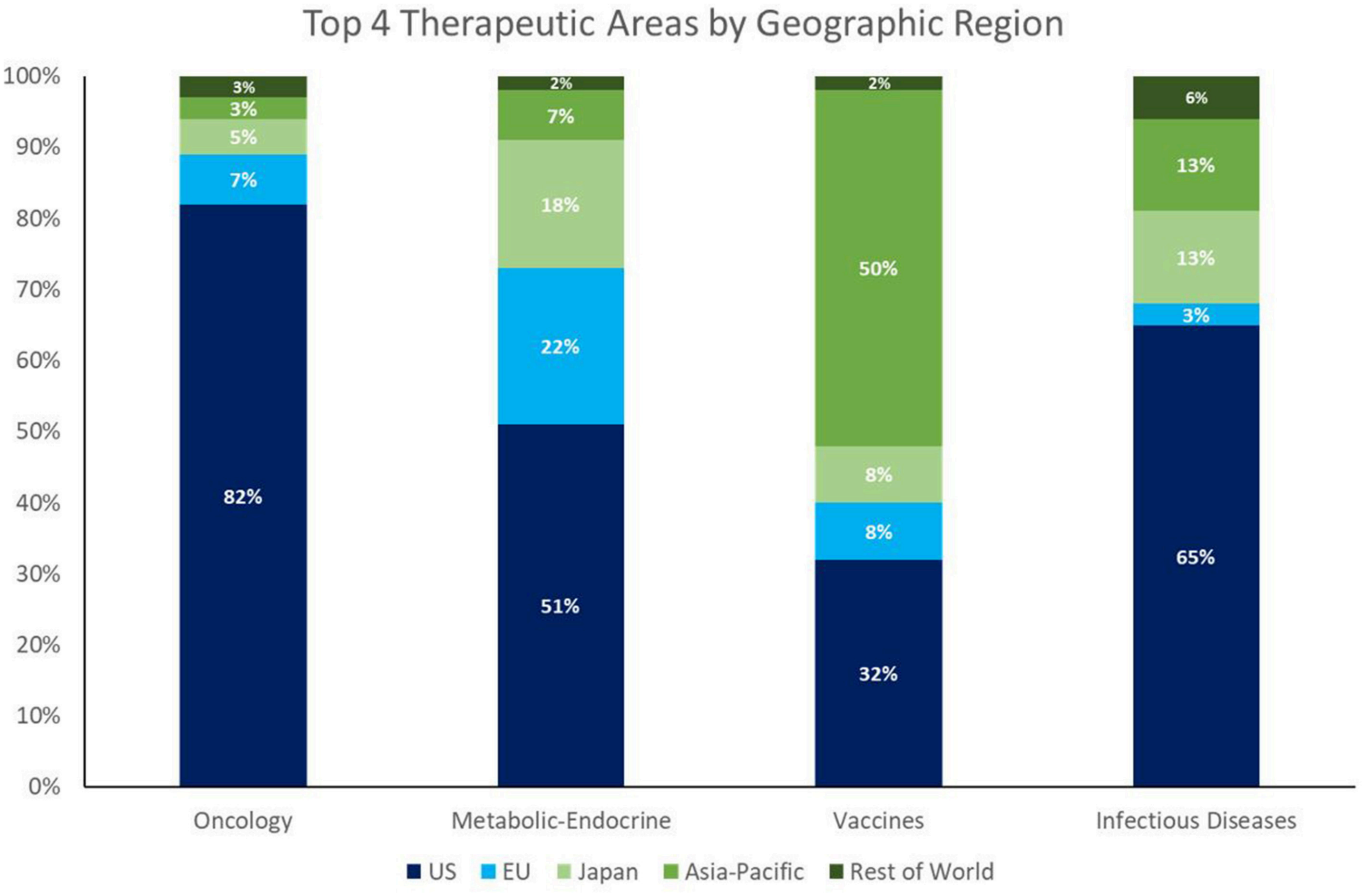
2013–2017 NAS, top 4 TAs by top 4 regions.

It is worth noting that the growth in NAS launches of ID products (both therapeutic and prophylactic) represents a positive trend and suggests an alignment of private/public resources and public health needs. This trend is the result of two factors. The first is that ex-US output of NAS appears to have a better balance of therapeutic areas than that of the US (see Figure 2). The second factor is that the pipeline investment in ID drugs has benefitted from strong public health advocacy—a type of advocacy fundamentally different from the patient-focused advocacy spearheaded by cancer patient organizations, such as the American Cancer Society, and those of other disease areas.

One example of the striking effectiveness of public health advocacy in ID is the creation of the Generating Antibiotic Incentives Now (GAIN) Act in the US, which resulted from the efforts of a stakeholder group of 50 healthcare and labor organizations, who petitioned the US Congress to address public health needs in the area of antibiotic resistance^4^. The GAIN Act allows for the expedited review and approval of new ID drugs, as well as 5 years of market exclusivity. The Act's effectiveness was highlighted in a 2017 US Government report, crediting the legislation with achieving 101 ID designations and six approvals <5 years into the program (GAO-17-189, 2017). Going forward, however, success in bringing new ID drugs to market is not guaranteed; it is dependent on FDA resources and political will.

## The Up And Down Sides of Facilitated Regulatory Pathways

The regulatory environment can have a sizeable impact on the introduction of innovative new medicines, especially in areas with high unmet medical needs but low market incentives. Whereas, the ability to set high prices for new drugs, and extend market exclusivity, act as “pull” incentives, in that they increase the likelihood of sufficient return on investment and spur new research and development (R&D) activity, regulatory initiatives aimed at speeding development and review times serve as equally powerful “push” incentives, in that they lower the financial and logistical barriers to market entry, and reduce the technical risk of product development (Milne, 2014).

The US FDA employs a full panoply of what are referred to as Facilitated Regulatory Pathways (FRPs), including (a) priority review (submissions receive a 6-month review time, compared to a 10-month standard review), (b) accelerated approval (conditional approval based on surrogate, or indirect measures of benefit), (c) fast track designation (increased access to scientific interaction with the FDA and rolling reviews of portions of product applications as they become ready), and (d) breakthrough therapy designation (BTD: includes fast track designation incentives and “all hands on deck” collaborative, cross-disciplinary engagement by the FDA).

Since 2000, oncology drugs have received 45% of all FRPs awarded by the FDA, representing 32% of all priority reviews, 53% of all accelerated approvals, and 50% of all fast track designations (Milne, 2014). This has contributed to industry's growing focus on oncology R&D, which has no doubt benefited from the expansive scientific knowledge base that exists due to the US National Institutes of Health (NIH) and academic medical centers' response in the 1970s to President Nixon's declaration of the “War on Cancer.” To highlight the point, during the decades of the 1980s and the 1990s, when cancer discovery efforts were still germinating, oncology drugs only represented 5 and 12% of overall US new drug approvals, respectively. By the first decade of the 2000s, however, that number reached parity with CV drugs at 19%. And in the period 2010–17, oncology drugs represented 29% of new approvals, compared to 14% for ID drugs, and 12% each for CV and CNS drugs^5^. In sum, in recent years, oncology drugs have been a major beneficiary of FRPs, which has stimulated investment in oncology R&D.

Is there a downside to FRPs? It is worth remembering that regulatory oversight is, in many ways, a zero-sum game. Political will and public advocacy are often lacking to address unmet medical needs in certain critical areas, and resources at regulatory agencies are finite. The US FDA itself has opined that such imbalances can result in boosted performance in one area to the detriment of another, effectively “squeezing out” certain therapeutic areas. There is a critical need for open debate to ensure alignment of public policy with public health needs.

## What Needs To Be Done

### Prioritization

Regional and national commissions should be created to review medical priorities, resource demands, and policy initiatives to achieve desired goals. Commissions should include experts from government, academia, industry, patient advocacy, insurers, and medical practice. The commissions should assess their region's immediate and long-term health needs and review the innovation landscape to determine whether current public and private R&D efforts are appropriately focused and funded.

Within regulatory authorities, FRP offices should be created to triage new drug applications. To help subsidize these activities, sponsors of candidate drugs could pay an application fee to the regulatory authority. If the FRP office determines that a drug candidate is eligible for one or more special regulatory programs, the sponsor would be exempt from paying any additional fees beyond standard user fees.

### Emerging Sponsors

The new drug research and development landscape is shifting dramatically, from the dominance of traditional big pharma to the emergence of venture capital–backed smaller companies and “emerging sponsors,” defined by the US FDA as the sponsor listed on the approval letter who is not a holder of a previously approved application. Sponsors are classified as “emerging” even if they have partnership or parent relationships with sponsors of a currently approved product. In recent years, ~40% of new drug and biologic approvals in the US were from emerging sponsors (Jenkins, 2012). Emerging sponsors share many of the same characteristics as start-up companies, in that they may have little or no experience with commercial drug development, the regulatory process, or product launch. Pharmaprojects reports that of ~4,000 pharmaceutical companies with active pipelines, 56% have just one or two products in the pipeline, tacitly qualifying them as emerging sponsors^c^. An FDA study documents that emerging sponsors are more likely to have multicycle reviews (DiMasi and Faden, 2009), and are less likely to garner approvals (50% approval rate as compared with 80% for medium/large companies) (Mathieu, 2013).

The relative lack of R&D experience of emerging sponsors highlights the need for institutional programs and courses that offer training in the drug development process. Several highly regarded programs currently exist, such as Tufts CSDD's Postgraduate Course in Clinical Pharmacology, Drug Development and Regulation; the IFAPP Academy-King's College London Medical Affairs in Medicines Development online course; the University of California: San Francisco's American Course in Drug Development and Regulatory Science; and the University of Basel's European Center for Pharmaceutical Medicine. These programs offer a broad yet comprehensive overview of the drug development and regulatory process.

### New Technologies

Oncology R&D has benefitted greatly from dramatic advances in our understanding of the immunologic and genetic bases of cancer. A majority of recently approved cancer drugs are considered among the most innovative genomically-targeted precision medicines. In the US, much of the growth in scientific knowledge can be traced directly back to a high number of research grants awarded by the National Institutes of Health that focus on immunology and cancer.

Despite remarkable advances in the oncology field, it is worth asking: In light of the increasing availability of prognostic and diagnostic technology available for CNS disorders, and promising new approaches in regenerative medicine to treat CVD, is the continued dominance of oncology/immunology out of balance with health needs, both economically and medically? According to Pharmaprojects, nearly 50% of the global R&D pipeline is focused on anti-cancer therapies (4232/8934 products in 2017)^c^. Some observers have suggested that this over-emphasis on oncology in global R&D pipelines is a misallocation of resources and has generated a surplus of competition in some relatively narrow cancer indications. Moreover, the likelihood of success for oncology product development is relatively low. In a 2016 analysis, SCRIP Pharma Intelligence determined that immuno-oncology is one of the least successful therapeutic areas in terms of Phase III projects moving on to a regulatory filing, with only a 40% transition probability, compared to 58% for all ~1,500 products included in the analysis (Lucy, 2016).

The US FDA, the EMA, and other national regulatory authorities have relied on regulatory science (i.e., developing new tools, standards, and approaches to assess safety, efficacy, quality, and performance) to understand and incorporate advances in new technologies. Nonetheless, challenges persist in agencies' attempts to integrate the risk-benefit profile of drugs, biologics, and devices during the product's entire time on the market. The goal is to close the evidence gap between the information regulators require to make decisions regarding product approval, and the type of information increasingly used by the medical community, payers, and others charged with making patient health care decisions.

### Global Competition vs. Harmonization

Asia (arguably excluding Japan) has been one of the greatest beneficiaries of globalization. The region as a whole accounts for 40% of world trade, according to the 2017 BCG report *How Asia Can Win in the new Global Era*. Recently however, some shifts in global economic currents have become detectable. Although manufacturing will remain an important contributor to growth in Asia, export-led economic models are now under pressure in most of the region. One reason for the decline is that trade, whose contribution to global GDP grew from around 25% in the 1960s to more than 60% in 2008, has since stalled. Another factor is that Asia's previously enormous manufacturing cost advantages have shrunk, as wage growth has outpaced productivity (BCG Henderson Institute, 2017).

Nonetheless, with 60% of the world's population, the Asia-Pacific region is a significant focus for pharmaceutical sales by both domestic and foreign firms. The region also appears poised to become a nexus for pharmaceutical production, especially for vaccines and generics. However, Asian policymakers and companies cannot rely excessively on export manufacturing. To remain competitive in the global marketplace and to meet the needs of its own burgeoning population, Asia-Pacific must nurture innovation, such as regenerative medicine, in research areas that offer promising advances for unmet medical needs through international collaboration, strategic partnerships, and global harmonization.

### Patient-Focused Drug Development

According to the US FDA, patient-focused drug development (PFDD) describes efforts to ensure that the review process benefits from a systematic approach to obtaining patient perspectives on disease severity and medical need. For example, in the CNS area, the FDA has proposed a new approach for Alzheimer's disease R&D that allows treatment of pre-symptomatic patients to slow the accumulation of substances in the body believed to be biomarkers of clinical disease, or to treat patients with early disease before functional impairment is apparent through an accelerated approval pathway on the basis of assessment of cognitive outcome alone. There is precedent for this type of PFDD from AIDS activism in the 1990s, during which the FDA and industry handled the risks through patient involvement in a meaningful process of informed consent (Powell, 2013).

For CNS drug development, in general, many major diseases and disorders may benefit from a PFDD approach. At a recent FDA meeting, patients with amyotrophic lateral sclerosis (ALS) argued emphatically that regulatory revamping is necessary to get research moving in the field, as there is only a single drug on the market for the disease (the orphan drug riluzole extends life of ALS patients by about 3 months). The ALS patients' recommendations were, in essence, a wish list for all unmet needs in CNS: (1) incentivize companies, in particular small companies that seem to populate this research area, by clarifying the regulatory pathway through guidance; (2) do not be overprotective of patients in terms of risk; (3) allow for abbreviated pre–investigational new drug toxicology testing; (4) permit the use of historical controls; (5) allow expanded access; (6) utilize accelerated approval; and (7) provide for a limited population designation under the guidance of supervising neurologists (Haley, 2013), as might occur under BTD.

Another condition that could benefit from a PFDD approach is obesity. The need was discussed at a George Washington University Stakeholder Panel in which it was suggested that obesity should be viewed as three conditions: obese but otherwise well; obese with risk factors; and obese and sick. In an Infectious Diseases Society of America approach, indications should be targeted to specific patient populations through Special Medical Use (SMU) designation to control off-label (and off-target) use, instead of risk evaluation and mitigation strategies (REMS), which were not designed for that purpose. Secondary end points should be added on the benefits side of the scale, such as effects on joint pain, urinary incontinence, sleep apnea, and mobility (Ferguson et al., 2013).

## The Way Forward

National regulatory authorities worldwide are responsible for protecting and promoting public health, yet they must often expend energy and resources reacting to public health emergencies and political pressure. They must engage with an increasingly global pharmaceutical enterprise, deal with growing patient activism, and leverage new technologies and social media, all the while remaining cognizant of national cost-containment pressures. Unfortunately, whereas the challenges have grown, the resources available to deal with them have remained the same or decreased. This disparity threatens to relegate the health problems that afflict the majority of patients at any given moment to secondary concerns. Innovation follows investment, and investors respond to the regulatory and economic climate. By continuing to emphasize PFDD, and by demonstrating regulatory flexibility in disease areas with high unmet need (beyond cancer, AIDS and orphan diseases), regulatory authorities can indirectly incentivize R&D in these important therapeutic areas.

There is no simple answer to how to stimulate innovation in therapeutic areas where the need is great but commercial incentives may be lacking. The solution requires a multi-stakeholder approach to identify demand and build consensus for change. Going forward, sponsors, regulators, policy makers, payers, academics, key opinion leaders, and, perhaps most importantly, patients, must work together to chart a course to a healthier future.

## Author Contributions

All authors listed have made a substantial, direct and intellectual contribution to the work, and approved it for publication.

### Conflict of Interest Statement

The authors declare that the research was conducted in the absence of any commercial or financial relationships that could be construed as a potential conflict of interest.

## Footnotes

^1^*Adapted from The Pharmaceutical Industry and Global Health: Facts and Figures 2012*, International Federation of Pharmaceutical Manufacturers & Associations.

^2^Note that in Figure 1, Metabolic-Endocrine, which appears as the second most common NAS therapeutic area, represents a composite category of drugs for endocrine diseases (e.g., type 2 diabetes), metabolic diseases, and congenital enzyme deficiencies (including many orphan drugs for rare conditions).

^3^*Decline and Fall of the Pharma Pipeline*. (2017). No. 3847. Available online at: https://scrip.pharmaintelligence.informa.com/SC098394/The-Decline-And-Fall-Of-The-Pharma-Pipeline (Accessed December 4, 2018).

^4^*50 Organizations' Letter to Congress on the Urgent Need for New Antibiotics*. (2012). Available online at: https://dpeaflcio.org/wp-content/uploads/Antibiotics-Sign-On-Letter-022212-House-Version.pdf. (Accessed November 19, 2018).

^5^*Unpublished Tufts CSDD Data*, Tufts CSDD Marketed Database, (2018).
